# Are there still indications for total pancreatectomy?

**DOI:** 10.1007/s13304-016-0388-6

**Published:** 2016-09-07

**Authors:** Marco Del Chiaro, Elena Rangelova, Ralf Segersvärd, Urban Arnelo

**Affiliations:** Pancreatic Surgery Unit-Division of Surgery, Department of Clinical Science, Intervention and Technology (CLINTEC), Karolinska Institutet at Center for Digestive Diseases-Karolinska University Hospital, K53-14186 Stockholm, Sweden

**Keywords:** Total pancreatectomy, Pancreas surgery, Arterial resection, Pancreatectomy

## Abstract

Total pancreatectomy is associated with short- and long-term high complication rate and without evidence of oncologic advantages. Several metabolic consequences are co-related with the apancreatic state. The unstable diabetes related to the total resection of the pancreas expose the patients to short- and long-term life-threatening complications. Severe hypoglycemia is a short-term dangerous complication that can also cause patients’ death. Chronic complications of severe diabetes (cardiac and vascular diseases, neuropathy, nephropathy, and retinopathy) are also cause of morbidity, mortality and worsening of quality of life. For this reasons the number of total pancreatectomies performed has certainly decreased over time. However, today there are still some indications for this kind of procedures. Chronic pancreatitis untreatable with conventional treatments, surgical treatment of precancerous pancreatic lesions, surgical treatment of locally advanced pancreatic cancer and the management of patients with extraordinary high-risk pancreatic texture after pancreaticoduodenectomy represent possible indications for total pancreatectomy and are analyzed in the present paper.

## Introduction

At the beginning of the era of oncologic pancreatic surgery, total pancreatectomy (TP) was used as a “radical procedure” for the surgical treatment of pancreatic cancer [1, 2]. However, new insights on pancreatic oncology, associated with the evidence of the side effects of total pancreatectomy, have made this procedure less appealing. The short- and long-term morbidity and mortality associated with the apancreatic state affecting these patients continue to be of concern today [3]. For these reasons, the complete removal of the pancreas is not considered today a more “radical” operation for patients suffering from pancreatic cancer and it cannot be considered the standard of care for the surgical treatment of these patients [4].

Even if the number of total pancreatectomies performed has certainly decreased over time, today there are still some indications for this procedure. Most of these are based more on local or personal experience rather than on true evidence and some data even show that the short- and long-term results of TP are not much different than undergoing a partial resection of the pancreas. A recent matched-pairs analysis of TP and pancreaticoduodenectomy, for example, revealed similar surgical outcome and long-term survival [5]. In the last decades, new advances in pancreatic surgery and in medical pancreatology have resulted in a new appraisal of TP as a treatment alternative for some pancreatic diseases or as technical option for some operative procedures.

In recent years, when major attention has been focused on preemptive pancreatic surgery [6], the role of TP has again been reevaluated. At the same time TP is proposed, with or without simultaneous auto-islet transplantation, for some types of chronic pancreatitis [7] and also for the treatment of locally advanced pancreatic cancer requiring arterial resection and reconstruction [8]. Total pancreatectomy combined with autologous islet transplantation has also been proposed for patients with a high-risk for complications resulting from the pancreatic anastomosis [9].

The aim of this review paper is to offer a complete overview of the outcome of TPs and to define the current indication for this procedure.

## Outcome of total pancreatectomy

### Surgical outcome

Generally, TP is considered a safer procedure than pancreaticoduodenectomy in the perioperative setting since a pancreatico-jejunostomy is not required. This implication is the basis for concept that TP may be an option for patients with a normal pancreas and a very high-risk for leakage after reconstruction, to prevent major postoperative complications. This theory has anyhow not been confirmed by the literature data. A recent analysis made on the US National Cancer Data Base [10] on 2582 patients who underwent TP for pancreatic cancer showed a 30-day mortality rate of 5.5 %. The same rate of short-term mortality was observed by Billings and co-authors [11] in a series of 99 consecutive TP, with the addition of 3 % of late death related to hypoglycemic episodes. The same authors investigated also the long-term QoL of 27 patients included in the study, showing a decrease of QoL compared with age- and gender-matched controls, but not compared to patients with diabetes from other causes. Even analyzing the recent data from the literature, it is quite evident that TP are associated with a significant postoperative morbidity and mortality, not inferior to partial pancreatectomy, as showed in Table 1 [5, 9, 10, 12–17].

**Table 1:** Short-term outcome of patients underwent TP

Author	Year	# Patients	# IAT	Overall morbidity (%)	Overall mortality (%)
Johnston PC	2016	2582	N.A.	N.A.	5.5
Balzano G	2015	28	28	57.1	7.1
Satoi S	2015	45	–	31.1	–
Chinnakotla S	2015	518	518	N.A.	9.2
Johnston PC	2015	18	18	50	N.A.
Wantabe Y	2015	23	–	43	4
Datta J	2015	64	–	45.3	1.6
Hartwig W	2015	434	–	37.6	7.8
Almond M	2014	80	–	46	12.5
Nikfarjam M	2014	15	–	87	8

### Metabolic consequences of total pancreatectomy

Several metabolic consequences are co-related with the apancreatic state, which characterizes the patients who underwent TP. Certainly, the most known and investigated sequel is diabetes. As defined by the American Diabetes Association, patients who underwent TP suffer from a type 3c diabetes, also called pancreatogenic diabetes [18]. In this form of diabetes, the glycemic control may be labile due to the loss of not only the insulin production, but particularly due to the loss of glucagon secretion [19]. The combination of insulin sensitivity and hypoglycemic unawareness, characteristic of this condition, is known as “brittle” diabetes [20]. It exposes patients who have undergone TP to short- and long-term life-threatening complications. In a recent series from Mayo Clinic, the large majority of patients undergoing TP (89 %) required a complex insulin regimen to maintain acceptable glycemic control. Seventy nine of them experienced episodes of hypoglycemia and 41 % of severe hypoglycemia. The resuscitation of these patients was possible by family members or co-workers only in 27 % of the cases, while the remaining 73 % required medical intervention or hospitalization [3]. Significant late mortality (3 %) due to hypoglycemic episodes has been reported by other authors [11]. The nonoptimal glycemic control is also the cause for chronic complications in the specific target organs. In the Mayo Clinic, the total cohort of patients who underwent TP experienced retinopathy in 3 %, neuropathy in 5 %, nephropathy in 4 %, cerebrovascular diseases in 1 %, cardiovascular diseases in 11 % [3].

The medical treatment of type 3c diabetes may be challenging. Insulin infusion pumps seem to improve the glycemic control and reduce the attacks of hypoglycemia [21]. However, evidence is lacking on the effect of this kind of treatment on the long-term complications of diabetes. The addition of glucagon injection to the insulin regimen seems to be advantageous in reducing the hypoglycemic episodes, but so far, only limited evidence about its usage is available [22].

Pancreatic exocrine insufficiency (PEI) is another consequence of TP. Even with high dose of pancreatic enzyme replacement therapy, a significant part of the patients who have undergone TP still suffer from steatorrhea, with consequent malabsorption, thus complicating further the already fragile management of diabetes [23]. PEI requires a lifelong substitutive treatment with pancreas enzymes [24]. However, the enzyme substitution in the majority of patients might not be enough to completely restore the normal digestion. A considerable number of the patients are thus undertreated for this problem, with underestimated and undiagnosed subclinical malnutrition even in the absence of steatorrhea [25]. Furthermore, alteration of the gastrointestinal motility is a well-known digestive disturbance associated with the development of severe exocrine insufficiency, but its role in post-pancreatectomy patients has not been investigated.

Liver steatosis is another complication considered to be associated with TP. It has been demonstrated that it may result in liver failure in some patients [26].

### Quality of life (QoL) after total pancreatectomy

QoL after TP is a very difficult topic to investigate and elaborate on. There are several and different instruments for the QoL measurement. The indications for TP and its associated procedures (i.e., auto-islet transplantation) are also several and different, as well as the initial condition of the patients and the expected long-term results and functionality, making it difficult to unequivocally apply all these instruments. In a recent small series Watanabe defined the QoL of a group on patients who underwent TP comparable to those of the national population, excluding for one third of the patients that experienced diarrhea [14]. Billings and colleagues [11] investigated the QoL of 34 patients who underwent total pancreatectomy with three different audit systems (SF-36, ADD QoL and EORTC PAN-26). They concluded that QoL after pancreatectomy is decreased compared with the age- and gender- matched controls, but not with diabetic patients for other causes.

## Indications for total pancreatectomy

### Chronic pancreatitis

Chronic pancreatitis (CP) is a progressive chronic inflammation of the pancreas that can be caused by several etiological factors. Currently, a step-up approach (medical treatment, endoscopic treatment, surgical treatment) for patients suffering from CP has been recommended to be the standard of care. The most common consequences of chronic pancreatitis are abdominal pain and exocrine and endocrine insufficiency. In case when the pain is associated with ductal dilatation, a decompressive procedure is indicated. In these cases surgery seems to be superior to endoscopy according to recent studies [27]. In the case of “small-duct” CP in patients resistant to medical treatment, TP with or without auto-islet transplantation has been proposed [28, 29]. The same indication is present for patients with hereditary pancreatitis who are at increased risk for cancer development.

TP with auto-islet transplantation has received increasing attention recently. The concept behind this approach is interesting, because the procedure seems to be safe and theoretically able to maintain good glycemic control, thus avoiding one of the devastating complications of TP [30]. The rate of insulin independence after auto-islet transplantation ranges between 24 and 40 % in the largest series as reported in Table 2 [28, 31–33]. For further, one third of the patients partial insulin dependence is achieved providing a better and more stable glycemic control. One of the most important predictor factors of insulin independence seems to be the quantity of the transplanted islets [28, 33]. For this reason, the timing of surgery for chronic pancreatitis is essential. Thus, in patients considered as candidates for TP with auto-islet transplantation, surgery should perhaps be considered earlier and certainly before the endocrine function is compromised. Furthermore, this procedure has shown even benefits in terms of the pain control. In other series, the range of pain-free or narcotic-independent patients is reported to be from 59 % to 80 % [28, 32]. The pain control seems to be stable over time, too. Ong and co-workers [31] reported that only 16 % of patients who underwent TP with auto-islet transplantation needed opiates at 5 years. For patients with chronic pancreatitis who have with maintained endocrine function but pain resistant to medical, endoscopic and surgical treatment, this option has shown also an improved quality of life [32].

**Table 2:** Outcome of total pancreatectomy + auto-islet transplantation for chronic pancreatitis

Author	Year	# Patients	Insulin independent (%)	Partial function (%)	Insulin dependent (%)
Sutherland DR	2012	409	30 (3 years)	33 (3 years)	37 (3 years)
Walsh RM	2012	20	20 (not specified)	–	80 % (not specified)
Ong SL	2009	50	40 (post-op)	–	–
Ahmad S	2005	118	40 (not specified)	–	60 (not specified)

### Premalignant lesions

TP has been proposed as a preemptive treatment for patients with a familial risk for pancreatic cancer [34]. Today, however, the prophylactic removal of the pancreas in individuals with a family history of pancreatic cancer is not recommended by neither national nor international guidelines [35, 36]. Rather, the treatment of detected precancerous lesions is advised, by partial pancreatic resections. However, unlike IPMNs, Pan-INs still cannot be detected by the current imaging modalities. This has raised the concern that undetected precancerous lesions might be left behind in the residual pancreas, thus leaning the choice towards TP. In case of abnormalities diffusely involving the pancreatic gland, in young patients and with high familial risk, though, a total pancreatectomy might be considered [37].

**Fig. 1:**
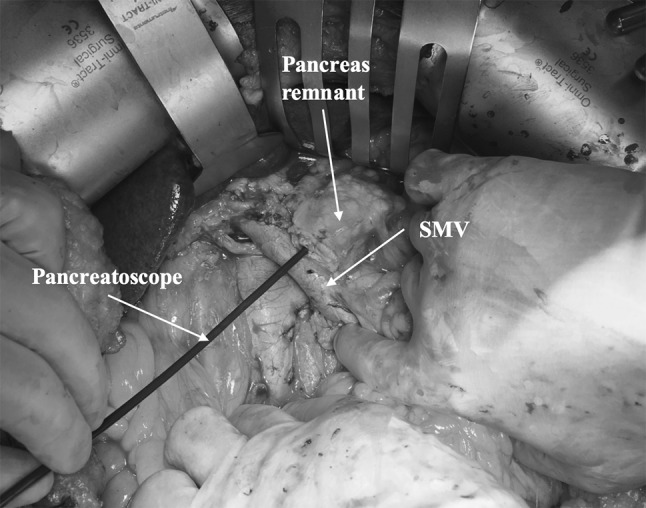
Picture of intraoperative pancreatoscopy. The pancreatoscope is inserted in the main pancreatic duct after the transection of the pancreatic neck in a pancreaticoduodenectomy procedure performed for main-duct IPMN. *SMV* superior mesenteric vein

Intraductal papillary mucinous neoplasms of the pancreas (IPMNs) also comprise precancerous lesions that potentially can progress into cancer. In accordance with the emerging literature data and with the European and International Guidelines, the majority of branch-duct IPMNs can be managed conservatively without the need for surgical resection [38, 39]. In contrast, the main-duct IPMNs require resection in every patient fit for surgery [38, 39]. There is emerging evidence in the literature supporting the indication for surgery even for relatively small dilatation of the main pancreatic duct, as recommended by the European Guidelines on Cystic Tumors of the Pancreas [40, 41]. When the dilatation of the duct is confined to a certain segment, a partial pancreatectomy with a frozen section of the resection margin is recommended [38]. The situation is more complicated when the entire duct is dilated. In this case, generally a pancreaticoduodenectomy with a frozen section of the resection margin is advocated to determine the extent of the resection [38]. The only scenario when total pancreatectomy is recommended is in case of diffusely extensive disease, with an advanced stage of dysplasia present in the entire duct. How reliable the frozen section is in determining the extent of the grossly dysplastic changes in the duct is questionable, and for sure, “skip lesions” of the main pancreatic duct can be missed. Pancreatoscopy, pre- or intraoperatively, can improve the accuracy of defining the extent of the disease and potentially better help select the candidates for a total pancreatectomy [42] (Fig. 1).

### Locally advanced pancreatic cancer

**Fig. 2:**
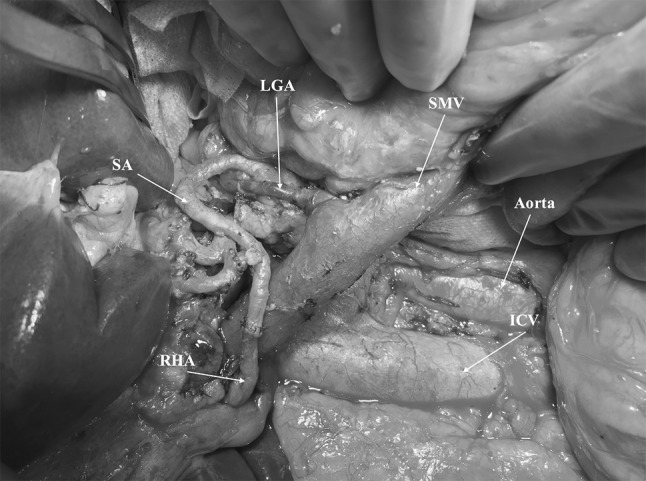
Intraoperative picture of total pancreatectomy associated with resection of the right hepatic artery (RHA) and reconstruction with the splenic artery (SA) rotated. *LGA* left gastric artery, *ICV* inferior cava vein, *SMV* superior mesenteric vein

Even if the surgical treatment of locally advanced pancreatic cancer is currently not the standard of care, it did receive more attention recently in the light of the more promising results of new neoadjuvant treatments [43]. In selected cases, surgery for locally advanced pancreatic cancer is also supported by the international scientific community [44]. When an arterial resection is required, the transposition or interposition of the splenic artery can, for instance, be used for the reconstruction both of the hepatic and superior mesenteric arteries [8, 46] (Fig. 2). In cases when arterial reconstructions are undertaken, TP is generally performed [8, 45]. The complete removal of the pancreas makes the procedure safer by eliminating completely the problem of pancreas fistula and it’s potentially fatal effect on the arterial anastomosis.

### High-risk pancreatic anastomosis

**Fig. 3:**
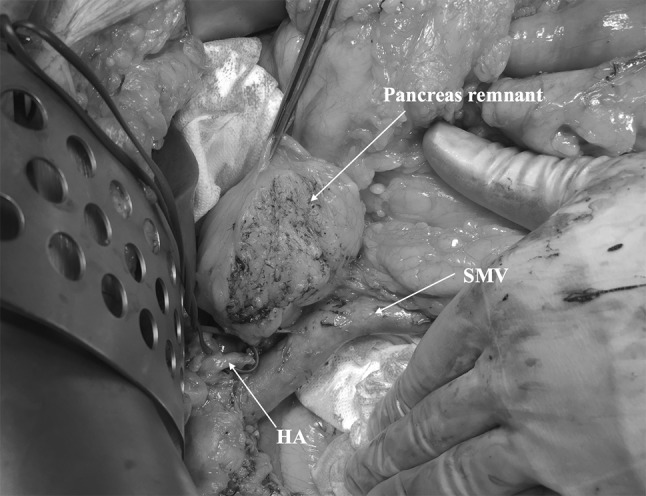
Intraoperative picture showing a totally fat-infiltrated pancreatic remnant after pancreaticoduodenectomy. *HA* hepatic artery, *SMV* superior mesenteric vein

A soft pancreatic texture and a small main pancreatic duct represent the major risk factors for the development of postoperative pancreatic fistula and these can easily be evaluated pre- and intraoperatively [47]. Overweight and pancreatic fat infiltration are other well-recognized predictors for the risk of postoperative fistula [48] and can as well create a technical problem in making the pancreatic anastomosis (Fig. 3). In selected cases of very high-risk patients, with a high-risk pancreas and other contributing risk factors, TP may represent an alternative to a pancreatic anastomosis in an attempt to reduce the significant postoperative morbidity and mortality. This approach has been proposed recently, even in a conjunction to auto-islet transplantation [9]. However, this decision should be always weighted against the risks associated with TP, as the postoperative mortality and morbidity (Table 1) of TP are significant as well as the long-term sequels of this operation [3].

## Discussion

In the last years, the number of the performed TP for the treatment of pancreatic diseases has been reduced in all high-volume pancreatic centers. There are several reasons for sustaining this trend. First of all, in oncologic surgery, there is no evidence that TP represents a more radical oncologic procedure [4]. [3]. In contrast, TP is associated with short- and long-term serious sequels and complications due to the “brittle” diabetes – 3 % late mortality caused by severe hypoglycaemia and chronic complications in the target organs.

However, even today, TP has its role in pancreatic surgery for some limited indications. As previously discussed, for patients affected by chronic pancreatitis, in whom the conventional medical and surgical approaches have failed, TP can represent the only therapeutic alternative, particularly, in association with auto-islet transplantation [10, 13] with encouraging results for pain, metabolic control and postoperative quality of life. The quantity of the islet cells and the timing of the procedure are crucial for the success of the auto-islet transplantation.

TP for the treatment of precancerous lesions has been decreasingly recommended as upfront approach, but rather partial resections, with intraoperative frozen section of the resection margin and/or pancreatoscopy to preserve pancreatic parenchyma [42]. However, in selected cases with diffuse dysplastic changes in the duct, TP still represents the best alternative for patients with main-duct IPMN.

In our experience, TP represents the best choice for patients who undergo pancreatectomy with arterial resections and reconstructions. The complete removal of the pancreas eliminates the risk of pancreatic fistula leading potentially to postoperative pseudoaneurysms and late erosive bleedings in the presence of simultaneous arterial anastomosis.

TP cannot be considered an equal alternative to pancreatic anastomosis to reduce the pancreas remnant complications, even with auto-islet transplantation, according to the current level of evidence in literature. However, in selected patients in poor general conditions, and with very high-risk pancreas and obese, TP may represent an adequate alternative technique [9].

In conclusion, even if the indications for TP have been consistently reduced over time, even today this procedure still has its place. The improvement of short- and long-term results of TP when combined with auto-islet transplantation may expand the spectrum of indication for this procedure in the near future.
